# Reactive lysine content in commercially available pet foods*

**DOI:** 10.1017/jns.2014.29

**Published:** 2014-09-30

**Authors:** Charlotte van Rooijen, Guido Bosch, Antonius F. B. van der Poel, Peter A. Wierenga, Lucille Alexander, Wouter H. Hendriks

**Affiliations:** 1 Animal Nutrition Group, Wageningen University, PO Box 338, 6700 AHWageningen, The Netherlands; 2 Laboratory of Food Chemistry, Wageningen University, PO Box 17, 6700 AAWageningen, The Netherlands; 3 Waltham Centre for Pet Nutrition, Freeby Lane, Waltham-on-the-Wolds, Melton Mowbray, LeicestershireLE14 4RT, UK; 4 Division of Nutrition, Faculty of Veterinary Medicine, Utrecht University, PO Box 80152, 3508 TDUtrecht, The Netherlands

**Keywords:** Maillard reaction, Minimal lysine requirement, Dogs, Cats, Nutritive value, ME, metabolisable energy, MLR, minimal lysine requirement, RL, reactive lysine, TL, total lysine

## Abstract

The Maillard reaction can occur during processing of pet foods. During this reaction, the ε-amino group of lysine reacts with reducing sugars to become unavailable for metabolism. The aim of the present study was to determine the reactive lysine (RL; the remaining available lysine) to total lysine (TL) ratio of commercial pet foods and to evaluate whether RL levels meet minimal lysine requirements (MLR). Sixty-seven extruded, canned and pelleted commercially available dog and cat foods for growth and maintenance were analysed for proximate nutrient composition, TL and RL. RL was expressed on a metabolisable energy basis and compared with the MLR for maintenance and growth. In dog foods, average RL:TL ratios were 0·87 (se 0·02) for extruded, 0·97 (se 0·02) for canned and 0·85 (se 0·01) for pelleted foods, with the lowest ratio of 0·77 in an extruded diet for growing dogs. In extruded and canned cat foods, the average ratio was 0·91 (se 0·02) and 0·90 (se 0·03), respectively, with the lowest ratio being 0·67 in an extruded diet for growing cats. Variation in the RL:TL ratio between and within processing type indicate that ingredients rather than processing might be the key factor influencing RL content in pet foods. Eight dry foods for growing dogs had RL contents between 96 and 138 % of MLR, indicating that RL has to be between 62 and 104 % digestible to meet the MLR. Considering the variability in RL digestibility, these foods could be at risk of not meeting the MLR for growing dogs. Ingredients and pet foods should be characterised with respect to the RL content and digestibility, to avoid limitations in the lysine supply to growing dogs.

Lysine is an essential amino acid for cats and dogs and sufficient amounts of bioavailable lysine need to be included in pet foods to meet the minimal lysine requirements (MLR) of these animals throughout their lives. Commercial pet foods vary greatly in format, ingredient and nutrient composition and type of processing. Thermal treatment is widely used in pet food production to improve digestibility of nutrients and to increase shelf life and food safety. Extruded kibbles are processed at temperatures of 80–200°C for 10–270 s under high pressure, while canned foods are heated until temperatures of 121°C for >10 min are reached at the centre of the can to sterilise the product^(^^1^^)^ and pelleted pet foods are relatively mildly heat-treated using temperatures of 60–90°C for 30–45 s^(^^2^^)^. Increased temperatures and residence times during processing are known to induce the Maillard reaction where a reducing sugar binds to a free reactive amino group of an amino acid, blocking the reactive site of the amino acid. When the reactive ε-amino group of lysine is the substrate of the reaction, modified lysine derivatives are formed, that may be partially absorbed but cannot be fully utilised by the animal, thereby, reducing the nutritive value of the food^(^^3^^–^^5^^)^. These derivatives can revert back to lysine under standard amino acid analyses conditions and, therefore, are assumed to be lysine molecules with a reactive ε-amino group. Direct determination of lysine with a free ε-amino group (reactive lysine (RL)) can be performed using the guanidination method, which uses O-methylisourea as a reagent to bind specifically. Recent studies using the guanidination method have reported that the difference between total lysine (TL) and RL of commercially produced canned and dry dog and cat foods can be considerable, with RL:TL ratio values reported as low as 0·38^(^^6^^–^^8^^)^. The residual RL appears not to be 100 % digestible either: standardised ileal O-methylisourea-RL digestibility values in dogs fed five commercial dry extruded dog foods were 79·5 to 93·7 % with a mean of 88·2 %^(^^9^^)^. Using the rat as a model animal, standardised ileal O-methylisourea-RL digestibility of canned cat foods ranged from 79·9 to 97·1 % with a mean of 88·1 %, whereas for the dry cat foods values ranged from 89·9 to 97·7 % with a mean of 94·8 %^(^^6^^)^. These results indicate that RL content in commercial pet foods can be reduced, possibly due to the Maillard reaction, and that RL content does not equal RL bioavailability.

Previous studies (e.g. Rutherfurd *et al.*^(^^6^^)^ and Williams *et al.*^(^^7^^)^) focused mainly on the difference between RL and TL contents of processed commercial pet foods. As these foods were not evaluated for metabolisable energy (ME) content, RL contents were not related to MLR in dog and cat foods. The objective of the present study was to determine the RL-to-TL ratio of commercial extruded, canned and pelleted pet foods to evaluate whether RL levels meet MLR for growing and adult dogs and cats as set out by the NRC^(^^10^^)^. Additional extruded junior foods were included to provide insight into the variability of RL content in the same brand/manufacturer junior foods with similar recipes, but produced in different countries over the world.

## Experimental methods

### Diets and sample preparation

A dataset was created containing 153 extruded, canned and pelleted pet foods commercially available in The Netherlands. Pet food types were categorised according to species (dogs or cats) and life stage (junior or adult). From each category in the dataset, five pet foods (single batch) were randomly selected. In addition, eleven extruded dry foods for growing dogs and six extruded dry foods for growing cats similar in recipe from a single manufacturer were obtained that were manufactured in Australia, Brazil, China, Germany, Mexico, Thailand, UK and the USA. This resulted in five commercial pellets, sixteen extruded and five canned foods for growing dogs; five commercial pellets, five extruded and five canned foods for adult dogs; eleven commercial extruded and five canned foods for growing cats and five commercial extruded and five canned foods for adult cats. According to the information on the labels of the junior foods, the food could be fed from the age of 4 weeks onwards. The moist canned foods were freeze-dried, and all the foods were ground to pass a 1 mm sieve in a Retch Mill (ZM100, Retch BV). All samples were stored in air-tight plastic containers at 4°C prior to analyses.

### Chemical analyses

DM and crude ash were determined by drying to a constant weight at 103°C (ISO 6496, 1999) and combustion at 550°C (ISO 5984, 2002), respectively. Crude protein (N × 6·25) was determined using the DUMAS method and crude fat was determined gravimetrically after hydrolysis with HCl and extraction with light petroleum (boiling point 40–60°C; ISO 6492, 1999). Crude fibre was determined gravimetrically as the remaining insoluble organic fraction after acid and alkaline digestion (ISO 6865, 2000). All analyses were performed in duplicate. ME content of the foods was calculated using predictive equations for ME^(^^10^^)^: digestible energy (DE, kJ) − (4·35 × g crude protein) for dogs and DE (kJ) − (3·22 × g crude protein) for cats; DE (kJ) = gross energy (GE, kJ) × energy digestibility (%)/100; energy digestibility (%) = 91·2 − (1·43 × % crude fibre in DM) for dogs and 87·9 − (0·88 × % crude fibre in DM) for cats; GE (kJ) = (23·85 × g crude protein) + (39·33 × g crude fat) + (17·15 × (g nitrogen-free extract + g crude fibre)); nitrogen-free extract in g/kg DM was calculated as 1000 – crude fat – crude protein – crude ash – crude fibre. Amino acids including TL were determined according to Hendriks *et al.*^(^^11^^)^ with O-methylisourea-RL determined according to Moughan & Rutherfurd^(^^12^^)^. RL was expressed on an ME basis and compared with the nutritional requirements for growth and maintenance of dogs and cats^(^^10^^)^.

### Statistical analyses

The effect of food type (i.e. extruded, pelleted and canned for dog foods, extruded and canned for cat foods) on RL:TL was tested for significance using ANOVA by Proc GLM of SAS 9.2 for Windows (SAS Institute, Cary, NC, USA). In case *P* ≤ 0·05 for significant effects, pairwise comparisons were made using *post hoc* analysis and corresponding *P* values were reported. Results are presented as the means with their standard errors .

## Results

**Fig. 1.**
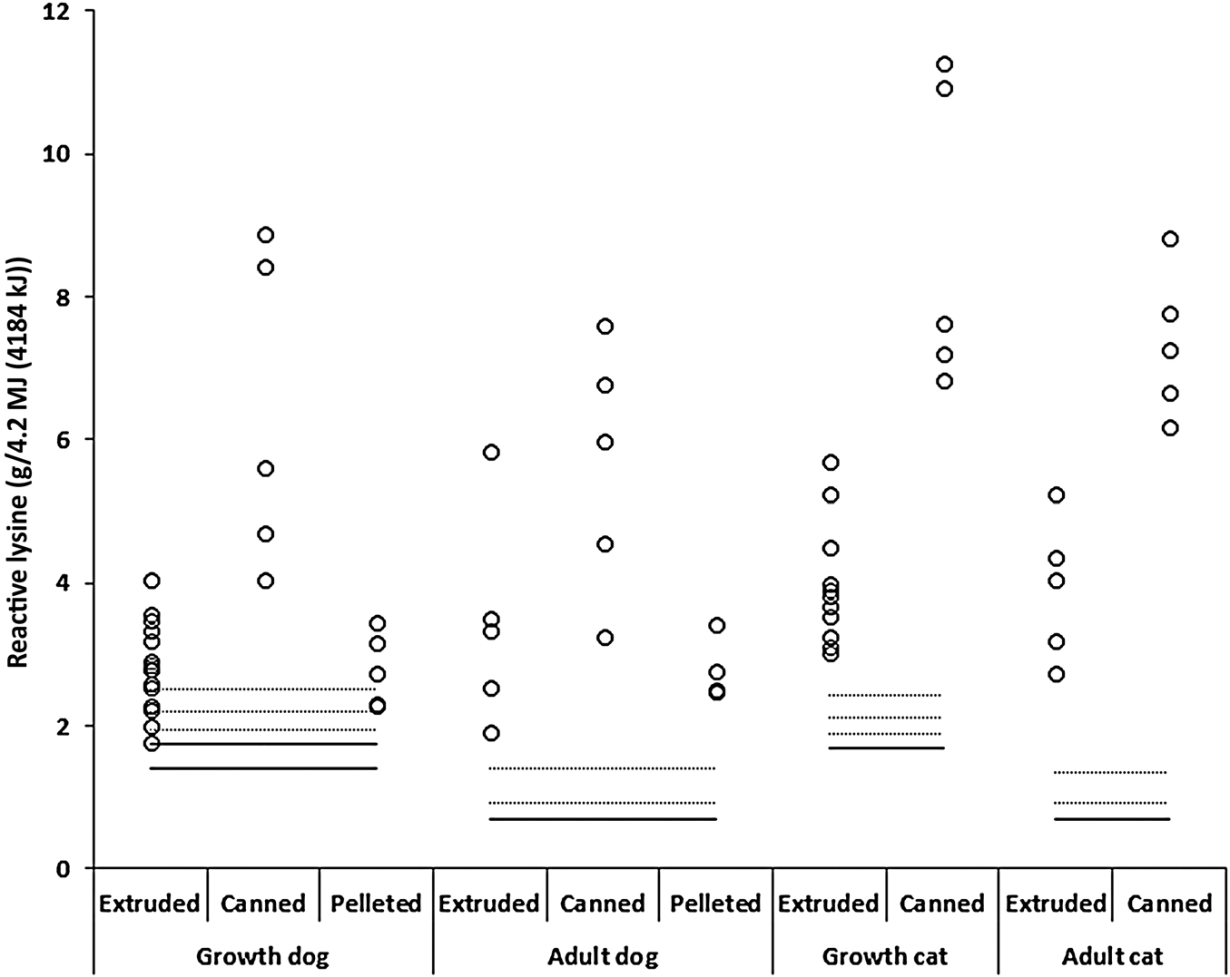
Reactive lysine contents of five commercial pellets, sixteen extruded and five canned foods for growing dogs; five commercial pellets, five extruded and five canned foods for adult dogs; eleven commercial extruded and five canned foods for growing cats and five commercial extruded and five canned foods for adult cats. Horizontal solid lines indicate minimum lysine requirements (MLR) for growing dogs between 4 and 14 weeks of age presented by NRC^(^^10^^)^ being 1·75 g/4·2 MJ (4184 kJ) ME (lower solid line), for growing dogs from 14 weeks onwards (1·40 g/4·2 MJ (4184 kJ) ME; upper solid line), for dogs at maintenance (0·70 g/4·2 MJ (4184 kJ) ME), for growing cats being 1·70 g/4·2 MJ (4184 kJ) ME and for cats at maintenance being 0·68 g/4·2 MJ (4184 kJ) ME. Dashed lines indicate bioavailability thresholds of 90, 80 and 70 % for meeting MLR of growing dogs between 4 and 14 weeks of age and growing cats, and of 75 and 50 % for meeting minimal lysine requirements for dogs and cats at maintenance.

Proximate analysis is shown in Table 1 and was on average for DM, crude protein, crude fat, crude fibre, crude ash, nitrogen-free extract (g/kg DM) and ME (MJ/kg), respectively: in extruded dog foods 921·97 (1·77), 300·40 (10·92), 153·27 (7·81), 19·67 (0·82), 74·05 (3·17), 452·61 (17·40) and 16·15 (0·16); in canned dog foods 217·77 (12·34), 497·61 (38·01), 22·96 (17·01), 13·01 (1·38), 102·84 (8·98), 172·22 (49·35) and 4·17 (0·26); in pelleted dog foods 907·40 (4·01), 260·79 (6·48), 123·45 (13·09), 23·55 (1·79), 78·92 (2·22), 513·29 (16·84) and 15·14 (0·28); in extruded cat foods 939·23 (3·38), 374·85 (11·33), 170·61 (10·08), 16·68 (1·38), 80·31 (3·86), 357·55 (18·78) and 16·79 (0·26); and in canned cat foods 210·98 (13·36), 660·09 (26·96), 243·00 (11·61), 9·71 (2·45), 102·12 (8·94), 34·70 (20·82) and 4·34 (0·30). In dog foods, average RL:TL ratios (Table 1) were 0·87 (0·02) (range 0·77–0·99) for extruded, 0·97 (0·02) (range 0·83–1·10) for canned and 0·85 (0·01) (range 0·78–0·94) for pelleted foods, with the lowest ratio of 0·77 in an extruded diet for growing dogs. Canned foods differed significantly from extruded (*P* = 0·004) and pelleted (*P* = 0·0008) foods. In extruded and canned cat foods, the average ratio was 0·91 (0·02) (range 0·67–1·03) and 0·90 (0·03) (range 0·75–1·03), respectively (*P* = 0·652), with the lowest ratio being 0·67 in an extruded diet for growing cats. RL:TL ratio in similar foods from countries around the world produced by the same manufacturer ranged from 0·77 to 0·94 in junior dog foods and 0·82 to 0·90 in junior cat foods. RL contents (g/kg DM) were: 14·5 (3·0), 24·2 (3·2) and 11·6 (0·8) for extruded, canned and pelleted adult dog foods, respectively; 13·7 (0·9), 28·4 (5·0) and 12·7 (0·9) for extruded, canned and pelleted junior dog foods, respectively; 16·4 (1·6) and 36·5 (2·5) for extruded and canned adult cat foods, respectively, and 17·4 (1·4) and 44·3 (3·7) for extruded and canned junior cat foods, respectively. The RL contents of all dog foods but one and all cat foods were higher than MLR for growing and adult cats and dogs as stated by the NRC^(^^10^^)^, ranging from 96 up to 581 % of the MLR (Fig. 1).

**Table 1.** Proximate composition, metabolisable energy (ME), total lysine (TL) and O-methylisourea-reactive lysine (RL) content, and RL:TL ratio of sixty-seven commercial pet foods categorised in animal species, processing type and life stage (means with their standard errors, g/kg DM unless defined differently)*

	Dog												Cat							
	Extruded				Canned				Pelleted				Extruded				Canned			
	Junior		Adult		Junior		Adult		Junior		Adult		Junior		Adult		Junior		Adult	
	Mean	sem	Mean	sem	Mean	sem	Mean	sem	Mean	sem	Mean	sem	Mean	sem	Mean	sem	Mean	sem	Mean	sem
DM^†^	921·9	2·0	922·3	4·2	231·3	18·0	204·2	16·4	909·3	5·6	905·5	6·3	939·5	4·2	938·6	6·4	240·7	18·4	181·2	4·8
Range	907·9–937·9		910·1–932·4		186·9–296·2		169·0–259·1		899·7–931·1		884·8–922·4		919·4–972·2		914·9–949·0		197·7–296·3		167·6–195·7	
Crude protein	301·5	8·7	297·0	39·8	512·6	57·4	482·6	55·6	260·3	9·6	261·3	9·9	393·6	12·3	333·6	9·2	700·8	30·2	619·4	39·1
Range	204·9–354·1		210·8–444·9		364·0–710·3		294·0–591·2		229·2–280·0		246·2–300·3		351·5–494·4		310·5–365·9		590·1–757·5		489·3–711·6	
Crude fat	160·0	8·6	131·6	15·7	224·0	26·7	221·9	24·2	154·7	12·1	92·2	11·6	166·8	11·8	178·9	20·8	246·6	15·5	239·4	19·0
Range	106·7–209·6		98·1–189·5		157·7–308·6		164·9–275·9		116·3–188·2		63·4–128·1		118·9–240·3		115·4–233·1		216·6–300·0		198·3–302·7	
Crude ash	73·8	3·8	74·8	6·2	104·6	9·0	101·0	16·7	78·1	3·0	79·8	3·6	82·6	4·5	75·4	7·6	90·3	14·8	113·9	8·4
Range	52·4–118·1		58·4–96·4		73·6–126·6		43·3–133·0		69·3–88·3		72·6–92·5		59·3–109·8		55·3–101·3		43·9–124·0		90·8–139·1	
Crude fibre	18·1	0·6	24·6	1·3	11·1	0·9	14·9	2·5	21·6	2·7	25·5	2·3	15·7	1·2	18·9	3·7	9·9	3·9	9·5	3·4
Range	15·3–23·5		22·2–29·4		8·2–13·5		11·3–24·5		12·2–27·7		21·1–32·9		7·6–21·6		11·7–28·7		1·5–24·1		3·9–21·9	
Nitrogen-free extract^‡^	446·6	14·8	472·0	60·0	163·1	55·9	181·3	88·3	485·3	21·0	541·3	21·0	341·3	22·5	393·2	23·4	19·2	19·2	50·2	38·3
Range	341·5–595·3		239·9–576·8		0·0–341·0		0·0–481·5		443·3–557·5		467·2–588·8		167·9–426·1		320·2–466·1		0·0–95·8		0·0–199·0	
ME^§^	16·3	0·2	15·6	0·3	4·5	0·4	3·9	0·3	15·8	0·3	14·5	0.2	16·8	0·3	16·9	0·6	5·1	0·4	3·6	0·1
Range	15·0–17·3		14·9–16·4		3·3–5·6		2·9–4·8		15·0–16·5		13·9–15·3		15·5–18·6		15·1–18·3		4·4–6·4		3·3–4·0	
TL	13·7	0·9	14·5	3·0	28·4	5·0	24·2	3·2	12·7	0·9	11·6	0·8	17·4	1·4	16·4	1·6	44·3	3·7	36·5	2·5
Range	8·1–16·6		9·1–26·0		19·4–44·9		16·3–32·8		10·6–15·7		10·2–14·4		15·5–28		12·5–20·2		36·2–55·1		36·2–55·1	
RL	11·9	0·9	13·2	2·8	27·1	4·5	23·9	3·6	10·9	0·8	9·9	0·8	15·0	1·1	15·9	1·8	40·7	4·9	32·1	2·2
Range	6·6–12·4		7·1–23·4		18·7–41·5		13·5–32·6		9·1–13·6		8·6–12·9		11·9–23·2		11·7–20·1		30·6–52·9		25·6–36·8	
RL:TL	0·87	0·02	0·90	0·03	0·96	0·02	0·98	0·04	0·86	0·02	0·84	0·01	0·88	0·03	0·97	0·02	0·91	0·05	0·88	0·02
Range	0·77–0·99		0·78–0·97		0·92–1·03		0·83–1·10		0·78–0·94		0·81–0·89		0·67–0·98		0·91–1·03		0·75–1·03		0·82–0·93	

* *n* 5, except for extruded junior dog foods (*n* 16) and extruded junior cat foods (*n* 11).

^†^g/kg.

^‡^Calculated as: 1000 – crude fat – crude protein – crude ash – crude fibre.

^§^MJ/kg; calculated using predictive equations for ME^(^^10^^)^.

## Discussion

The variation in RL:TL ratio observed in the present study (Table 1) between, as well as within the diet types (i.e. extruded, canned, pelleted) may be due to: (1) the use of different processing conditions, (2) different durations of drying and storage and (3) the use of different ingredients^(^^13^^)^. The low RL:TL ratios in pelleted foods compared with extruded or canned foods is in line with the result of Tran *et al.*^(^^8^^)^, in which pelleted diets, on average, contained a lower RL:TL ratio (0·80; range 0·72–0·93) compared with extruded diets (0·87; range 0·83–0·93). These results are unexpected as pelleting is generally carried out at lower temperatures compared with extrusion. Considering extruded and canned pet foods in the present study, the average and minimal RL:TL ratio of extruded dog foods (0·87; range 0·77–0·99) was lower compared with canned dog foods (0·97; range 0·83–1·10); however, for the cat foods the results were comparable (0·90, range 0·67–1·03 for extruded *v.* 0·90, range 0·75–1·03 for canned cat foods). The results of the present study were higher compared with previous studies. Rutherfurd *et al.*^(^^6^^)^ reported average RL:TL ratios of 0·51 (range 0·38–0·61) for canned cat foods and of 0·59 (range 0·51–0·80) for dry cat foods. Williams *et al.*^(^^7^^)^ reported average RL:TL ratios of 0·85 (range 0·44–1·06) for adult dog foods, and of 0·75 (range 0·47–0·98) for foods for growing dogs. Although other factors such as drying and storage can reduce the RL content of extruded diets^(^^14^^,^^15^^)^, the unexpected results for the pelleted foods, as well as the discrepancy between results for extruded and canned dog and cat foods, indicate that ingredient choice, and not processing type, can have a major influence on RL:TL ratio.

Ingredients used in recipes for pelleting often include pre-treated ingredients. Carbohydrate sources for example, are often pre-treated as pelleting processing temperatures and residence times are not high enough to fully gelatinise the starch in the raw ingredients during pelleting^(^^16^^)^. The production of meat meal, used in pelleted and extruded foods, includes rendering under high temperatures. Rendered meals generally show a lower protein quality compared with raw animal meals, measured using lysine availability in a chicken growth assay^(^^17^^)^. RL:TL ratio in animal protein sources ranged from 0·64 to 0·99 and in vegetable protein sources from 0·56 to 1·00^(^^13^^)^. Therefore, it is likely that the loss of RL already starts at the selection of ingredients included in the pet food recipe; however, future studies measuring RL:TL ratio in ingredients and final product testing would need to be carried out to further investigate this hypothesis.

MLR reported by NRC^(^^10^^)^ relate to highly (95–100 %) bioavailable lysine. Although most of the dog and cat foods contained RL contents higher than MLR for growing and adult dogs and cats as stated by the NRC (Fig. 1)^(^^10^^)^, actual digestibility and subsequent availability of the RL in practical diets could be lower than the bioavailability assumed by the NRC^(^^10^^)^. Apparent ileal crude protein digestibility has been shown to be highly variable among 141 experimental dog foods, with values ranging from 51·1 up to 90·5 % with a mean digestibility of 73·5 %^(^^18^^)^. Variability in amino acid digestibility is not fully comparable, but it can be expected to be similar to crude protein digestibility. Therefore, these results indicate that lysine digestibility is likely to also vary. As indicated above, standardised ileal RL digestibility in pet foods (dry and moist) ranged between 79·5 and 97·7 %^(^^6^^,^^9^^)^, showing that lysine available to the animal is lower than the RL content.

The reduced lysine availability compared with the RL content might be of particular significance during growth. Growing dogs have a lower gastric pepsin secretion compared with adult dogs, which may result in digestibility values lower than those observed in adult dogs^(^^19^^)^. In the present dataset, eight dry (pelleted and extruded) foods for growing dogs between 4 and 14 weeks of age had RL contents between 96 and 138 % of MLR, indicating that RL has to be between 62 and 104 % digestible to meet the MLR. Considering the variability in RL digestibility, these foods could be at risk of not meeting the MLR for growing dogs. These results are comparable with the dataset used by Williams *et al.*^(^^7^^)^, that included two out of fourteen commercial foods for dogs between 4 and 14 weeks of age which had an RL content below MLR, and one food needed an RL digestibility of more than 90 % to meet MLR^(^^13^^)^. Dry foods for growing cats and adult dogs and cats, as well as canned foods contain RL contents that meet MLR if the RL digestibility of these foods is above 55 %.

In conclusion, variation in RL:TL ratio is high between and within processing types, and ingredients rather than processing or storage may be a key factor influencing the RL:TL ratio in processed pet foods. Foods for growing dogs that are used as weaning diets could be at risk of not meeting MLR, depending on the digestibility of RL. Ingredients and pet foods should be characterised with respect to the RL content and digestibility, to avoid limitations in the lysine supply to growing dogs.
